# Cotton Leaf Spot Detection Based on an Improved YOLOv11n Model

**DOI:** 10.3390/jimaging12070284

**Published:** 2026-06-27

**Authors:** Yaxin Xie, Mingyu Zhang, Yonghua Han, Le Dai, Haifeng Fu, Lu Xu

**Affiliations:** School of Information Science and Engineering, Zhejiang Sci-Tech University, Hangzhou 310018, China; 2023336711011@mails.zstu.edu.cn (Y.X.); 2024331200212@mails.zstu.edu.cn (M.Z.); 2023329600141@mails.zstu.edu.cn (L.D.); 2023332871016@mails.zstu.edu.cn (H.F.); xulu@zstu.edu.cn (L.X.)

**Keywords:** cotton disease detection, YOLOv11n, small object detection, ghost convolution, adaptive feature fusion

## Abstract

In cotton disease detection, the complex farmland environment and the varying scales of disease spots, especially the presence of small-target disease spots, limit the detection accuracy of lightweight models. To address this issue, an improved YOLOv11n detection algorithm is proposed. First, the backbone network is reconstructed using the GhostConv (G-conv) module, which generates redundant feature maps through linear operations, thereby reducing computational complexity. Second, an Adaptive Calibration and Feature Fusion Architecture Head (ACFFA) with prior calibration and cross-scale fusion capabilities is constructed in the detection stage to handle the problem of varying disease spot scales. Furthermore, the Adaptive Scale-aware Wise Intersection over Union (AS-WIoU) loss function, improved from WIoUv3, is introduced to enhance the stability of bounding box regression and improve detection accuracy for low-resolution, small-target lesions. Experimental results show that on the cotton disease dataset constructed based on the Mendeley Data database, the proposed model achieves *mAP*50 and *mAP*50-95 of 90.30% and 73.84%, respectively, with precision and recall of 92.33% and 87.68%, and a parameter count of 3.81 M. The algorithm significantly improves detection accuracy while maintaining efficient inference, making it suitable for real-time monitoring tasks on agricultural embedded terminals.

## 1. Introduction

Cotton, as an important economic crop globally, is not only the main natural fiber source for the textile industry but also has significant importance in biofuel production and oil processing [1,2]. The stable development of the cotton industry directly affects the global agricultural trade balance and the sustainability of related industries.

However, during growth, cotton is susceptible to infections by fungi, bacteria, viruses, etc., which in turn triggers the outbreak of various foliar spot diseases such as Blight, Curl, and Grey Mildew, leading to problems like reduced cotton yield and deteriorated quality, causing serious losses to growers [3]. These types of diseases often present different symptoms on plant leaves. Therefore, developing a fast and accurate system for identifying cotton leaf diseases has become an urgent need [4].

Before the rise of computer vision technology, the detection of cotton diseases could be divided into three categories: artificial visual observation, laboratory pathological analysis, and early instrumental detection techniques. Visual observation, as the most basic detection method, relies on agricultural technicians manually observing the color and morphological changes in leaves, stems, and bolls to determine the type of disease, or through systematic field surveys to record and analyze the obtained data systematically [5]. When visual symptoms are unclear, researchers often turn to laboratory pathological analysis to accurately classify pathogens through microscopic morphological identification, pathogen culture, and serological testing [5]. In addition, some researchers use instruments such as spectrometers to detect changes in cotton leaf reflectance, or use quality analyzers to manually inspect the color, length, and impurity content of cotton fibers to distinguish disease types [6]. These traditional cotton disease identification methods heavily rely on manual labor, often accompanied by problems such as high time consumption and strong dependence on professional knowledge, and are highly subjective, making them prone to missed detection and misjudgment of early-stage disease spots [7].

The development of digital image processing technology has opened a new path for crop disease detection. Early automatic detection of cotton diseases mainly relied on manual feature extraction and classical machine learning algorithms. Revathi and Hemalatha performed threshold segmentation in the CMYK color space to extract features, achieving preliminary disease spot classification [8]. Patil et al. built an early recognition system for cotton foliar spot diseases by integrating multi-feature extraction such as color, shape, and texture, Sobel edge detection, and support vector machine (SVM) classification [9]. Rumel M S Pir et al. used K-means clustering for image segmentation and a backpropagation neural network (BPNN) to classify the extracted features, constituting a cotton foliar spot disease recognition system [10]. Although these traditional machine learning methods have a certain degree of objectivity, manually designed features limit their generalization ability and robustness, making it difficult for the models to cope with interferences such as strong light, leaf occlusion, and complex backgrounds [11].

Facing the demand for real-world field identification, researchers have turned to deep learning techniques that can automatically learn multi-dimensional features. Convolutional neural networks (CNNs), with their excellent nonlinear features, bring high robustness, and the object detection architectures based on them have achieved a leap from classification to localization [12,13]. Two-stage algorithms such as Faster R-CNN significantly improve the localization accuracy of tiny disease spots through region proposal networks (RPNs), but their complex inference processes make them unsuited to meet the requirements for embedded deployment [14]. Thus, the YOLO series has emerged due to its end-to-end prediction mechanism and fast inference speed [15].

To meet the specific requirements of cotton disease detection, researchers have carried out multi-dimensional deep improvements on the YOLO architecture. Shao et al. introduced a coordinate attention mechanism into YOLOv5, significantly enhancing the model’s spatial awareness [16], thereby improving the accuracy and robustness of cotton disease identification in complex environments. Li et al. used modules such as CFNet to perform structural enhancement on YOLOv8s, improving the extraction precision of cotton disease and pest features [17]. To further capture early-stage ambiguous disease spots, Xu et al. introduced a global attention mechanism into YOLOv8 to build long-range dependencies, effectively improving the capability to capture sparse targets [18]. Meanwhile, CM-YOLO, proposed by Abudukelimu et al., further ensured the completeness of feature information through a multi-directional scanning strategy [19].

In addition to lightweight CNN detectors, end-to-end object detection methods based on Transformers, such as DETR [20], have also provided new research directions for plant disease detection. To address the shortcomings of slow convergence and insufficient small-target detection capability of DETR, Deformable DETR introduces a multi-scale deformable attention mechanism [21]; furthermore, RT-DETR is further oriented toward real-time detection tasks, incorporating an efficient hybrid encoder and a query selection mechanism, improving inference speed while maintaining the advantages of end-to-end detection [22]. In recent years, methods such as WMC-RTDETR and ToT-Net have also applied RT-DETR and its improved variants to agricultural disease detection tasks, aiming to enhance detection performance under complex backgrounds, occluded targets, and multi-scale disease spots [23,24]. However, Transformer-based detectors typically require large amounts of training data and high computational resources, with relatively complex model structures and cumbersome training and hyperparameter tuning. For cotton disease detection tasks, disease spots in field images are often small in scale, have blurred boundaries, and exhibit limited visual differences between categories. Directly deploying complex Transformer detectors can easily lead to high inference latency, weak generalization ability, and difficulties in practical deployment.

Furthermore, hybrid architectures that integrate CNNs and Transformers are gradually becoming a new trend in the field of plant disease detection. CNNs possess strong capability in extracting local texture, edge, and color features, while Transformers excel at modeling long-range dependencies and global context. The combination of the two can, to a certain extent, balance local disease spot details with the overall leaf structure, significantly enhancing the model’s feature representation capability in complex scenarios [24,25]. However, hybrid architectures inevitably increase network complexity and the number of hyperparameters, leading to higher training costs, more complicated ablation analyses, and greater difficulty in practical deployment. Especially on agricultural mobile devices with limited computational resources, how to introduce global modeling capability while controlling model size and inference cost remains a key issue that needs to be addressed.

In recent years, feature fusion strategies in plant disease detection have gradually evolved from traditional hierarchical fusion structures such as FPN and PANet to more sophisticated approaches including dynamic weighting, multi-scale context modeling, attention-guided fusion, and cross-scale adaptive interaction. To address the issues of small lesion scales, blurred boundaries, irregular shapes, and susceptibility to complex background interference, YOLOv11-MSDFF-RiceD designs an efficient multi-scale feature fusion module (EMFFM) that enhances the representation capability of disease features at different scales through partial channel fusion and residual connections, while reducing parameter count and memory usage [26]. YOLO-MSCM introduces a multi-scale context spatial attention module and a SimRepHMS module to enhance local context utilization and multi-scale feature fusion, thereby improving crop disease detection performance in complex environments [27]. In addition, MSFF-DETR constructs a multi-scale feature fusion network based on an improved RT-DETR, combining a multi-scale dilated residual module with a multi-scale feature pyramid network to improve information transfer efficiency across different feature levels under complex backgrounds [28]; AF-RT-DETR further enhances cross-scale feature interaction and suppresses redundant information through a bidirectional cross-gating mechanism, reflecting the development trend of feature fusion strategies in real-time Transformer detectors [29]. However, although the above methods improve the model’s adaptability to multi-scale lesions and complex backgrounds, the complex fusion structures often increase model design difficulty, computational cost, and deployment overhead. Meanwhile, excessive introduction of shallow features may bring noise information such as leaf veins, shadows, and background textures, leading to feature redundancy or cross-scale feature conflicts. Therefore, how to enhance the representation capability of small-target cotton disease features while controlling model complexity, and improve the model’s real-time performance and generalization ability in natural field environments, remains a problem to be further addressed in cotton disease detection research.

Although the above architectures have certain advantages in cotton disease detection, there is still room for improvement in terms of computational redundancy, multi-scale lesion capturing capability, and the identification of low-resolution, small-target disease spots. To this end, this paper proposes an improved model based on YOLOv11n. The core differences from the existing work are as follows.

Compared with the above studies, the core difference in our method does not lie simply in introducing more complex attention mechanisms, Transformer structures, or multi-branch fusion networks. Instead, it lies in a systematic and synergistic design centered around three core bottlenecks in real-world cotton disease detection: lightweight feature extraction, multi-scale lesion fusion, and low-quality small-target localization.

First, unlike existing methods that enhance feature representation by stacking attention modules, dense scanning mechanisms, or global modeling units, this paper adopts a design strategy of “standard convolution for high-fidelity perception in the first layer + Ghost convolution for redundancy suppression in subsequent layers” in the backbone network. On one hand, it preserves shallow details such as texture, color, and edges of the original disease spots; on the other hand, it reduces the redundant computation caused by deep standard convolutions, thereby achieving a balance between basic feature quality and lightweight deployment requirements under complex field backgrounds.

Second, unlike RT-DETR-like models or CNN-Transformer hybrid architectures that mainly rely on global attention modeling to enhance cross-scale perception, the proposed ACFFA module adopts a combination of channel prior calibration, pixel-wise cross-scale weighting, and dilated convolution detail refinement to achieve multi-scale lesion feature alignment without introducing heavy Transformer encoders. This design pays more attention to the common issues in cotton disease spots, such as blurred boundaries, scale variations, and shallow-level noise interference, and can avoid feature conflicts caused by the direct fusion of excessive shallow background textures.

Finally, unlike general bounding box regression losses such as CIoU, DIoU, and WIoU, the proposed AS-WIoU further introduces a target-scale dynamic awareness and boundary blur metric mechanism, enabling the model to dynamically adjust regression weights according to lesion scale and sample quality. This makes it particularly suitable for localization tasks involving early-stage small lesions, low-resolution images, and complex backgrounds. Therefore, our method is not a simple superposition of existing modules, but a structured improvement targeting the specific challenges of computational redundancy, cross-scale semantic conflicts, and low-quality small-target localization in real-world cotton disease detection.

Based on the above design rationale, the main contributions of this paper are as follows:

1. To address the issues of redundant computation and channel overlap in traditional convolutional layers, a lightweight backbone network based on G-conv is proposed. The first layer retains standard convolution to capture core receptive field features, while subsequent layers replace standard convolution with G-conv, thereby significantly reducing parameter overhead while maintaining rich feature representation. This achieves a balance between efficient inference and basic feature quality in resource-constrained environments.
2. To address the spatial information loss caused by downsampling and multi-scale semantic conflicts, ACFFA, a feature repair and fusion network, is proposed. Channel prior calibration is used to achieve cross-layer feature alignment, and dilated convolution is employed to compensate for edge details of tiny disease spots, thereby effectively improving the model’s ability to recognize multi-scale disease spots.
3. To address the issues of annotation noise interference in complex backgrounds and the limited recognition accuracy of low-resolution, small targets, an adaptive loss function, AS-WIoU, is designed. It adopts a target area threshold filtering mechanism and a blur-aware weight factor to dynamically evaluate sample quality, endowing the model with the ability to autonomously judge image quality, and is also applicable to instance segmentation and rotated bounding box detection tasks.

## 2. Materials and Methods

For different application scenarios, the YOLOv11 architecture provides five parameter configurations from Nano to Extra Large according to computational scale [30]. Considering that farmland monitoring equipment has high requirements for inference speed, the lightest version in this series, YOLOv11n, is selected as the baseline platform to be improved.

### 2.1. Overview of YOLOv11n

**Figure 1:**
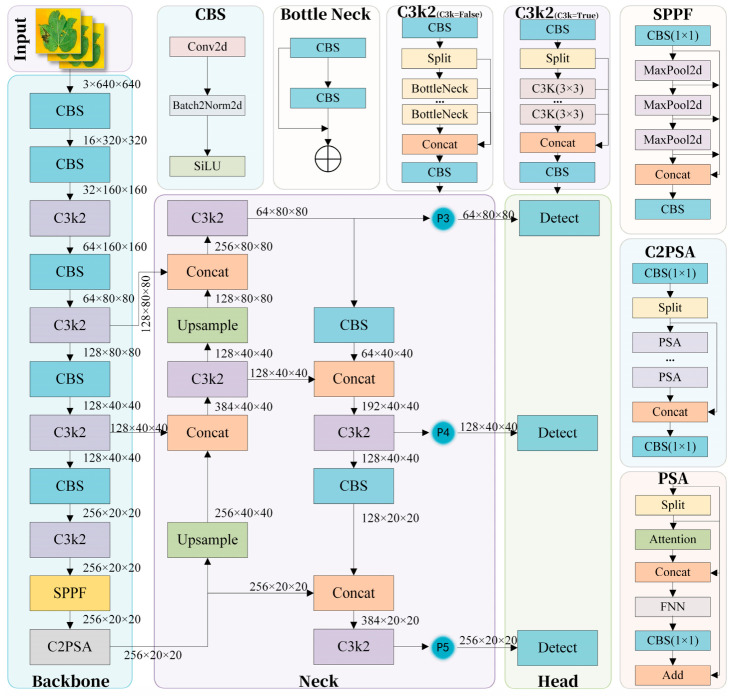
YOLOv11n architecture diagram.

In 2024, Ultralytics continuously absorbed and integrated the latest deep learning technologies and launched the YOLOv11n open-source general-purpose model [30]. While inheriting the basic framework of YOLOv8, it optimizes computational efficiency and cross-task versatility, further improving detection accuracy, inference efficiency, and robustness, and enhancing its adaptability to complex environments and high real-time scenarios [31,32]. YOLOv11n follows the classic three-stage architecture of backbone, neck, and detection head, with each part optimized. Its specific structure is shown in Figure 1.

The core optimization of the YOLOv11n backbone lies in the introduction of C3k2 and C2PSA [33]. Compared with C2f, C3k2 balances parameter efficiency and feature extraction capability by simplifying residual connections and adjusting convolutional kernel configurations [34]. On this basis, the C2PSA spatial attention module is added at the end to focus on key features [35]. This enables YOLOv11n to capture subtle features more accurately while maintaining real-time performance, providing high-quality feature maps for subsequent fusion and enhancement modules. Its neck network continues to use the PAN-FPN structure, integrating deep semantic information and sensitive shallow positional information through a bidirectional adjustment propagation mechanism, thereby enhancing the model’s multi-scale target detection capability. Compared with other versions of the YOLOv11 series, YOLOv11n’s PAN-FPN reduces the number of channels entering the neck network and, together with C3k2, flexibly scales the channel numbers in the PAN-FPN structure, increasing the information density of feature fusion while reducing computational cost [36]. The detection head of YOLOv11n continues the decoupled head architecture, separating classification and regression tasks, and adopts an Anchor-Free strategy to achieve detection by predicting the target center point and boundary offsets. To reduce computational load, YOLOv11n replaces some ordinary convolutions with depthwise separable convolutions, balancing accuracy with real-time requirements [37].

In summary, YOLOv11n is capable of capturing subtle, multi-scale features in real time, making it suitable as a baseline platform for the detection task of cotton foliar spot diseases. Facing issues such as diverse lesion morphologies, blurred boundaries, and dense distribution in field environments, this architecture can support edge device deployment while meeting the analytical requirements of complex pathological features as much as possible.

### 2.2. The Improved YOLOv11n Model

#### 2.2.1. Overview of the Improved YOLOv11n Model

Although YOLOv11n combines real-time performance with strong analytical capability, it still suffers from certain feature redundancy and has limitations when handling low-quality samples such as cross-scale disease spots, complex field environments, and early pathological features [38]. To address the three issues of computational redundancy in the basic feature extraction stage, weight imbalance in the cross-scale feature fusion process, and insufficient robustness of bounding box regression to noise and small targets, the following improvements are proposed.

First, the traditional backbone network of YOLOv11n is improved. Traditional backbone networks typically rely on deep convolutional architectures to capture fine disease spot textures and complex contextual semantics. However, stacking a large number of standard convolutions leads to severe parameter redundancy, resulting in increased inference latency. On the other hand, simply reducing the number of channels can easily cause the loss of low-level spatial information, thereby weakening the model’s ability to capture early tiny lesions. To address this, the improved model introduces G-conv to reconstruct the backbone network, aiming to maintain rich features while improving computational efficiency.

Second, the feature fusion method of YOLOv11n is improved. In the detection scenario of cotton foliar spot diseases, the contributions of features from different levels to the final prediction are often not equal. The original YOLOv11n model only integrates high-level semantics and low-level details through feature concatenation. This static and equal fusion method ignores the interrelationships and complementarity among features. When cotton leaves are severely overlapped and disease spot sizes vary dramatically, the model fails to fully utilize features, leading to issues such as false positives, missed detections, and severe degradation of boundary segmentation [39,40]. To address the above challenges, an adaptive channel fusion module ACFFA is proposed.

Finally, the loss function is improved. Limited by factors such as detection equipment and environmental conditions, training samples often come with certain annotation noise due to issues like imaging jitter, insufficient illumination, or cluttered backgrounds. Meanwhile, the default loss function of YOLOv11n adopts a consistent penalty strategy for all training samples, which tends to cause overfitting in the middle and late stages of training when dealing with early tiny disease spots or targets with blurred boundaries, leading to degraded localization accuracy [41]. Therefore, a loss function AS-WIoU with the capability of fuzzy boundary recognition and area awareness is developed.

**Figure 2:**
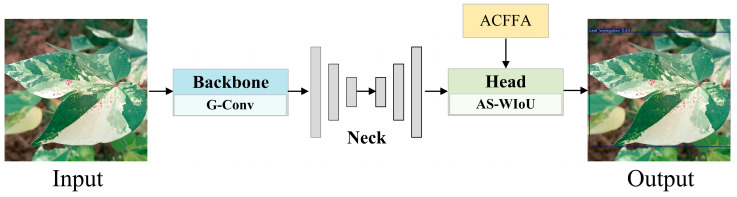
Schematic diagram of the improved model structure.

The schematic diagram of the improved structure of the final enhanced cotton foliar spot disease recognition model based on YOLOv11n is shown in Figure 2. 

**Figure 3:**
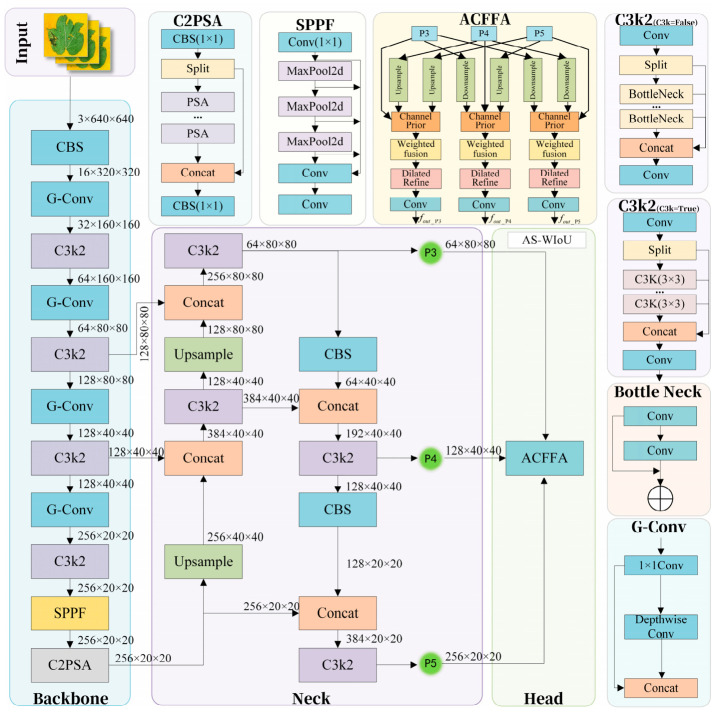
Schematic diagram of the improved YOLOv11n model architecture.

The detailed structure of the improved model is shown in Figure 3.

Compared with the original YOLOv11n, the improved model replaces all standard convolutions in the backbone network with G-conv modules except for the first convolutional layer. In the detection head, the self-designed adaptive channel fusion module ACFFA is introduced. Meanwhile, the AS-WIoU loss function optimized based on WIoUv3 is adopted to perform the bounding box regression localization task.

#### 2.2.2. A Lightweight Backbone Network Based on Ghost Convolution

The backbone network is responsible for extracting high-dimensional abstract features from raw pixels. Traditional deep convolutional neural networks (CNN) obtain the receptive field by stacking a large number of standard convolutional layers. Although this improves the robustness of the model to a certain extent, the resulting feature maps often exhibit high similarity, leading to a waste of computational resources.

**Figure 4:**
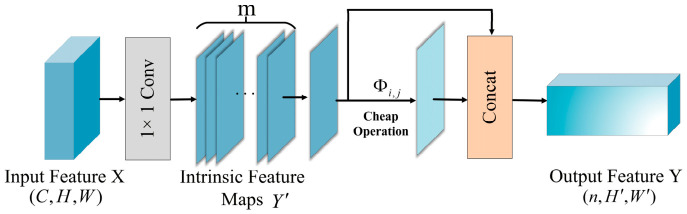
Diagram of the ghost convolution (G-conv) module.

To this end, a lightweight backbone network structure based on G-conv is proposed. To intuitively illustrate the implementation process of the lightweight mechanism, Figure 4 shows the internal architecture and feature evolution logic of G-conv.

Unlike standard convolution, which performs full computation for all output channels, G-conv decomposes the mapping process into two stages. First, a small number of intrinsic feature maps are generated using standard convolution. Let the input feature be $X\in R^{C\times H\times W}$, and the output be n channel feature maps, where C denotes the number of input feature channels, H and W denote the height and width of the input feature, respectively. The product of these three quantities appearing in subsequent formulas has the same meaning. To obtain n channel feature maps, the intrinsic feature maps $Y^{\prime }$ are first computed using mathematical Formula (1):

(1) $$Y^{\prime }\;=\;X\;*\;K,$$

where ∗ denotes the convolution operation, $K\in R^{C\times k\times k}$ is the convolution kernel, with a total of m kernels, each having C channels and a size of k×k pixels. The output is $Y^{\prime }\in R^{m\times h^{\prime }\times w^{\prime }}$, where m < n and n is an integer multiple of m, with the multiple factors being s. Subsequently, to restore the complete feature space, G-conv performs n − m linear transformations $\Phi_{i,j}$ on the obtained intrinsic feature maps $Y^{\prime }$ to generate replica feature maps, as expressed mathematically in Formula (2):

(2) $$y_{ij}=\Phi_{i,j}(Y_{i^{\prime }}),\;\forall i\in \{1,\ldots ,m\},\;j\in \{1,\ldots ,s-1\}.$$

Here $y_{ij}\in R^{(n-m)\times h\times w}$, and $y_{i}^{\prime }$ is the i-th channel of the intrinsic feature map Y’ given by Formula (1). m is the number of convolution kernels used in generating the intrinsic feature map. As mentioned earlier, s=n/m, then $n\;-\;m\;=\;sm\;-\;m\;=\;m\;\left(s-1\right)$. Thus, j denotes the j-th channel of the replica features generated from the i-th intrinsic feature map channel, and $\left(s-1\right)$ denotes the number of replica feature channels generated from each intrinsic feature map channel. $\Phi_{i,j}$ represents a linear operation, which can be implemented using depthwise separable convolution. Finally, the intrinsic features with m channels and the generated replica features with $m\left(s-1\right)$ channels are concatenated along the channel dimension to form a complete output feature Y with n channels. Through this design, under the same input and output dimensions, the computational acceleration ratio $r_{s}$ of G-conv compared to ordinary convolution can be approximately expressed as:

(3) $$r_{s}=\frac{n\times k\times k\times C}{m\times k\times k\times C+(n-m)\times d\times d}\approx \frac{s\times C}{C+s-1}\times \frac{k^{2}}{d^{2}}\approx s,$$

where s has the same meaning as in Formula (2), representing the number of linear transformations; n is the output dimension, i.e., the number of channels of the output feature; m is the number of channels of the intrinsic feature map generated by Formula (1); k is the size of the ordinary convolution kernel; and d is the size of the convolution kernel used in the replica feature generation in G-conv, with d = k. When the number of input feature map channels C of the G-conv module is much larger than $\left(s-1\right)$, Formula (3) holds. At this point, the computational cost is reduced by a factor, reserving some computational budget for subsequent computationally intensive modules.

Considering that the initial layer of the backbone network is primarily responsible for capturing high-frequency details of the image, directly applying lightweight operators would easily cause severe truncation of low-level spatial information. Therefore, the first convolutional layer of the backbone network is retained as a standard convolution to ensure high-fidelity perception of the original signal, while all subsequent deep feature extraction stages are replaced with G-conv. This allows the model to achieve a significant reduction in parameter count while still providing highly discriminative feature benchmarks for downstream modules.

#### 2.2.3. Design of Adaptive Calibration and Feature Fusion Architecture Head (ACFFA)

Cotton disease spots have various morphologies and a wide range of scales, making it difficult for models to effectively extract and represent multi-scale features. To address this issue, starting from the problems of channel repair and cross-scale semantic gaps in the fusion process, an adaptive channel feature fusion detection head ACFFA is designed, inspired by the Adaptively Spatial Feature Fusion (ASFF) architecture [42].

**Figure 5:**
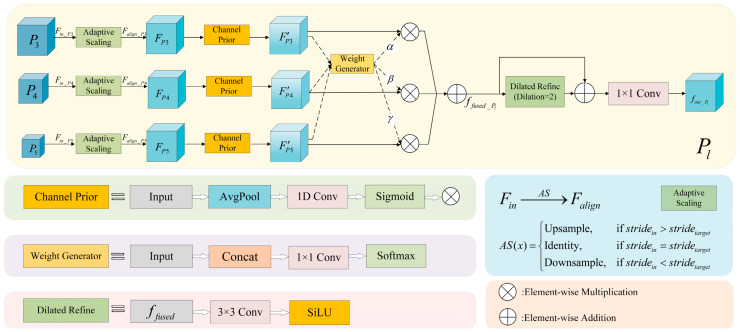
Schematic diagram of the operation logic of the Adaptive Calibration and Feature Fusion Architecture (ACFFA) module at the $P_{l}$ layer.

ASFF first adjusts feature maps of different depths to a unified scale through interpolation (upsampling) or strided convolution (downsampling) to eliminate the inherent scale inconsistency of feature pyramids. It then performs element-wise weighted summation on the aligned features using adaptively learned spatial weights, thereby achieving effective fusion of multi-scale features. However, this fusion method still has certain limitations: the weighting logic of ASFF essentially only focuses on the spatial dimension at a single level, ignoring the discriminative ability of the channel dimension in representing disease spot semantic information. Meanwhile, traditional linear interpolation and downsampling operations, when adjusting resolution, are prone to geometric distortion and aliasing of feature edges, often leading to loss of key information at critical locations when dealing with fine textures such as cotton disease spots. ACFFA provides targeted optimizations for the above issues, and its operation logic is shown in Figure 5.

For any layer (denoted as $P_{l},l\in \{3,4,5\}$) in Figure 3, ACFFA first receives the feature maps from $P_{3},P_{4},P_{5}$ all three layers. Then, according to the resolution of the current target layer $P_{l}$, it dynamically aligns the feature maps of the other two layers to the size of $P_{l}$ using upsampling or downsampling. If the input layer is larger than the target layer, downsampling is performed; otherwise, upsampling is used to expand the scale. All inputs are unified to the same scale $F_{P_{l}}$, establishing a spatial benchmark for subsequent processing.

After scaling to a uniform size, due to the significant differences in semantic information and background noise among the feature maps $P_{3},P_{4},P_{5}$ at different depths of the feature pyramid, direct fusion often leads to dilution of effective features. To address this, a channel prior calibration component, denoted as “Channel Prior’’ in Figure 5, is introduced in the design of ACFFA. This component first compresses the spatial information of each channel of the three-scale feature maps after size unification using global average pooling, extracting a one-dimensional channel descriptor C×1×1 that represents global semantics. Then, a 1D convolution is used to achieve local cross-channel interaction while preserving the integrity of the channel structure. Subsequently, a Sigmoid activation function is introduced to map the calibrated values to the interval (0,1) to generate accurate channel weight masks. Finally, the weights are reapplied to the original feature maps through element-wise multiplication to obtain weighted three-dimensional vectors. This process dynamically adjusts the weights according to channel contributions. After training, it can enhance channels that are highly correlated with disease spot color and texture, and suppress irrelevant noise signals caused by illumination fluctuations, leaf overlap, or soil background, thereby providing a better feature benchmark for subsequent spatial weight calculation. This channel calibration mechanism can be simplified into the mathematical expression shown in Formula (4):

(4) $$F_{p_{l}}^{\prime }=\sigma \left(\mathrm{Conv}1d\left(\mathrm{AvgPool}(F_{p_{l}})\right)\right)⊗F_{p_{l}},\;l\in \{3,4,5\},$$

where σ represents the Sigmoid activation function, and ⊗ denotes element-wise multiplication along the channel dimension.

After channel calibration, ACFFA adopts an adaptive weighting strategy to resolve the semantic conflicts in cross-scale integration. As shown in the middle part of Figure 5, the calibrated features $F_{P_{i}}$ are fed into the weight generator to obtain two-dimensional vector weights α, β, and γ for the spatial pixel points of $P_{3}$, $P_{4}$, and $P_{5}$ at the $P_{i}$ layer. This component first concatenates the feature maps of each scale along the channel dimension to construct a feature (3C,H,W) that incorporates multi-receptive field information. Then, a 1×1 convolution is used to perform cross-scale spatial correlation mapping for each pixel position while maintaining the original spatial resolution, obtaining pixel-level raw scores for each scale. Finally, Softmax normalization is applied along the scale dimension to convert the raw activation values output by the convolution into weight scores, resulting in a weight map of size (3,H,W), where the three channels of the weight map correspond to α, β, and γ in order. The three weights and the original feature maps are linearly weighted summed to obtain the final feature fusion map. For level $P_{i}$, the final fusion output can be expressed as:

(5) $$f_{\mathrm{used}\_P_{l}}=\alpha \times F_{P_{3}}^{\prime }+\beta \times F_{P_{4}}^{\prime }+\gamma \times F_{P_{5}}^{\prime },\;l\in \{3,4,5\},$$

where α+β+γ=1, i.e., the sum of the corresponding points of the three two-dimensional vectors α, β, and γ, equals 1. This mechanism enables the network to increase the weight of high-resolution shallow features in the edge regions of diseased tissues to preserve accurate boundary gradients, while in the core regions for determining disease spot categories, it tends to rely on deep semantics with a broader receptive field. This approach effectively mitigates the semantic cancellation problem in multi-scale feature integration and ensures the precision of feature representation.

During cross-scale feature alignment, the resampling operation introduces inevitable geometric distortion and edge blurring. To address this, ACFFA integrates a repair branch based on dilated convolution at the output stage. As shown in the Dilated Refine module in Figure 5, considering that cotton foliar spot diseases have relatively uniform color and similar spatial sampling point features, this component adopts a 3 × 3 convolution with a dilation rate of 2 to expand the receptive field while avoiding excessive detail loss during channel information fusion, thereby effectively alleviating geometric distortion. Subsequently, batch normalization and the SiLU activation function are cascaded to ensure training stability. On this basis, the repair branch adds the above output to the original fused features through a residual structure, and finally, a 1 × 1 projection layer is used to achieve channel alignment and refinement, further addressing the edge blurring problem. The process is shown in Formula (6):

(6) $$f_{\mathrm{out}\_P_{l}}={\mathrm{Conv}}_{1\times 1}(f_{\mathrm{fused}\_P_{l}}+\mathrm{DilatedConv}(f_{\mathrm{fused}\_P_{l}})),\;\;\;\;l\in \{3,4,5\}.$$

This repair mechanism allows the model to use a broader neighborhood context around the disease spot to perform secondary correction of pixel responses, sharpening the damaged edge details of the disease spot. This strategy ensures that the output feature $f_{out_{P_{l}}}$ is highly aligned with the actual physical disease spot in terms of geometric morphology, thereby improving the model’s detection robustness for cotton foliar disease spots with irregular shapes and blurred boundaries.

#### 2.2.4. Design of Adaptive Scale-Aware Wise Intersection over Union (AS-WIoU)

**Figure 6:**
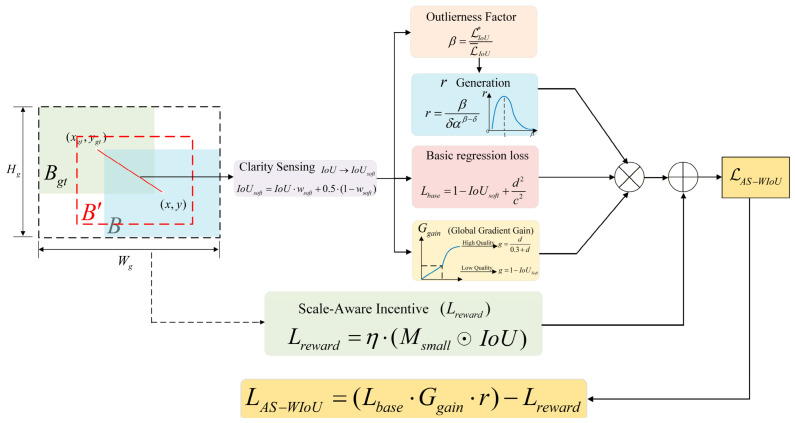
Structural diagram of the Adaptive Scale-aware Wise Intersection over Union (AS-WIoU) mechanism.

In cotton leaf disease detection, disease spots are extremely small in the early stage, and the complex field environment combined with imaging and recognition limitations leads to blurred edges. To address these two issues, the AS-WIoU loss function is constructed based on WIoUv3. This design introduces an adaptive adjustment mechanism with area awareness and clarity awareness. This mechanism enables the model to autonomously judge sample quality and dynamically adjust regression weights and learning strategies. Its structure is shown in Figure 6.

The spatial mapping relationship between the predicted box B and the ground truth box $B_{gt}$ as shown in Figure 6 is the underlying logic of the loss function. AS-WIoU first establishes the basic geometric loss $L_{IoU}=1-S_{inter}/S_{union}$ based on the intersection over union (IoU) criterion in the Cartesian coordinate system, where $S_{inter}$ is the intersection area between the predicted box B and the ground truth box $B_{gt}$, and $S_{union}$ is the union area of the two boxes. $L_{IoU}$ is used to quantify the spatial overlap degree between the two boxes B and $B_{gt}$, while also providing a primitive reference benchmark for the subsequent introduction of nonlinear gains.

Considering that in complex natural field environments, imaging blur often leads to severe gradient noise in the regression target, traditional hard intersection-over-union metrics are unable to provide stable guidance at uncertain edges. To address this, as shown in Figure 6, AS-WIoU constructs a clarity-aware mechanism. This mechanism transforms the original metric into a soft metric with error tolerance capability through nonlinear mapping, and its mathematical expression is as follows:

(7) $$IoU_{soft}=IoU\times w_{soft}+0.5\times (1-w_{soft}),$$

where $w_{soft}$ is a manually preset soft weight coefficient that determines the system’s tolerance for uncertain boundaries. The system reconstructs the basic regression cost $L_{base}$, outlier factor β, non-monotonic focusing coefficient r, and global gradient gain $G_{gain}$ through $IoU_{soft}$, thereby affecting the overall loss function.

To construct the above $L_{base}$, the Euclidean distance d between the center points of the minimum enclosing area of the predicted box B and the ground truth box $B_{gt}$, as well as the diagonal length c of the minimum bounding rectangle, are first obtained. Then, the regression cost function shown in Formula (8) is constructed:

(8) $$L_{\mathrm{base}}=1-IoU+\frac{d^{2}}{c^{2}}.$$

Next, based on Formula (8), AS-WIoU integrates a dynamic focusing mechanism to address the gradient jitter caused by varying fitting difficulties of training samples. This mechanism introduces an outlier factor $\beta =L_{IoU}^{*}/\bar{L_{IoU}}$, as shown in Figure 6, where $L_{IoU}^{*}$ represents the real-time IoU loss of the current single sample, and $\bar{L_{IoU}}$ is the moving average loss that rolls with the training process. β is used to evaluate sample quality in real time. Then, to achieve scientific gradient allocation, a non-monotonic focusing coefficient $r=\frac{\beta }{\delta \alpha^{\beta -\delta }}$ is computed based on β to dynamically scale the regression gradient, where α and δ are manually set adjustment parameters used to cooperatively control the adjustment mechanism for samples of different difficulty levels.

To further stabilize the training process, as shown in Figure 6, the system introduces a global gradient gain $G_{gain}$ to adaptively adjust the regression strength. This factor implements piecewise stimulation based on the IoU value of the sample: for high-quality anchor boxes with IoU greater than a preset threshold $\tau_{blur}$, the system uses a nonlinear gain g=d/(0.3+d) constructed from the deviation d=|IoU−0.7| to compress gradient fluctuations; for low-quality anchor boxes below the threshold, the geometric deviation g=1−IoU is directly used as the feedback benchmark. Through this differentiated global gain design, the model is able to maintain gradient stability and efficiency at different training stages.

To address the problem of information loss of extremely small targets in deep feature maps at the early stage of disease, AS-WIoU simultaneously designs an area-aware mechanism. This mechanism monitors the physical pixel area of the target and actively identifies tiny disease spots whose area is smaller than a preset threshold $A_{min}$. To compensate for the insufficient gradient contribution of such targets in backpropagation, the system introduces an incentive for small targets:

(9) $$L_{\mathrm{reward}}=\omega_{\mathrm{reward}}\times (M_{\mathrm{small}}⊙IoU),$$

This incentive term is jointly driven by a manually preset coefficient $\omega_{reward}$ and a small target mask $M_{small}$, providing additional gradient gain when the model successfully covers tiny disease spots, thereby improving the model’s ability to capture early subtle symptoms.

Combining the aforementioned basic regression cost $L_{base}$, global gradient gain $G_{gain}$, non-monotonic focusing coefficient r, and small target incentive $L_{reward}$, through the operations of ⊗ (denoting element-wise multiplication) and ⊙ (denoting element-wise addition) as shown in Figure 6, the complete loss function of AS-WIoU is finally constructed, as shown in Formula (10):

(10) $$L_{\mathrm{AS-WIoU}}=L_{\mathrm{base}}\times G_{\mathrm{gain}}\times r-L_{\mathrm{reward}}$$

This loss function enables the system to output robust regression gradients when facing cotton diseases under different growth stages and imaging conditions, thereby further improving the localization accuracy of the model.

## 3. Results and Discussion

### 3.1. Data Acquisition and Preprocessing

To construct a cotton leaf disease recognition dataset oriented toward complex farmland environments, this study selected the public dataset [43] from the Mendeley Data open repository as the core data foundation and performed systematic screening and structural optimization on the original field images. To alleviate the class imbalance issue after sample filtering, an additional open-source dataset [44] was further introduced for cross-supplementation. The benchmark dataset [43] contains approximately 2137 cotton leaf images, covering seven categories: Curl Virus, Healthy, Leaf Hopper Jassids, Leaf Redding, Leaf Variegation, Bacterial Blight, and Herbicide Growth Damage. Among them, about 46.05% of the images were captured under controlled laboratory conditions, characterized by uniform background colors (e.g., pure white, pure black, or uniform blue screens), even illumination controlled by artificial light sources, leaves flattened or fixed on uniform platforms, and the absence of natural field interferences such as soil or other leaves. To improve the model’s generalization ability in real farmland scenarios, samples under controlled environments were systematically removed according to the above criteria, resulting in the removal of 984 images. Among them, all images of the Herbicide Growth Damage category were captured under controlled environments. Moreover, considering that this category corresponds to non-infectious chemical injury rather than pathogen-induced disease, it was entirely excluded from the dataset. After the above screening, the sample sizes of Bacterial Blight and Healthy were relatively small, leading to class imbalance. To alleviate this issue to some extent, another open-source dataset [44] was further introduced as a supplementary source. After screening according to the same field-scene criteria as the primary dataset, merging, and deduplication, a final set of 1224 original images was obtained.

Subsequently, the X-AnyLabeling platform was used to manually annotate the above original images. The annotation strategy in this study is differentially defined based on the phenotypic characteristics of the diseases. This annotation strategy aligns with the actual manifestation of field diseases and aims to enable the model to learn more generalizable lesion representations. The specific rules are as follows:

1. Whole-leaf annotation: applicable to systemic diseases or large-area infection-type diseases, such as Curl Virus, which manifests as overall leaf curling, deformation, and yellowing; Leaf Redding, which presents as extensive reddening of the leaf; and Leaf Variegation, which shows mottled yellow-green discoloration across the entire leaf. The pathological characteristics of such diseases cover the whole leaf; therefore, the bounding rectangle of the entire leaf is used as the annotation unit.
2. Single-lesion annotation: applicable to discrete disease spots with clear boundaries, such as Bacterial Blight, which manifests as polygonal necrotic lesions confined by leaf veins. Such lesions have distinct boundaries and can be individually distinguished; therefore, the bounding rectangle of each single lesion is used as the annotation unit.
3. Block annotation: applicable to small and dense spot-like symptoms, such as Leaf Hopper Jassid damage, which appears as tiny yellowish-white spots scattered on the leaves. Such lesions are numerous and individually small; annotating each one individually is neither economical nor necessary. Therefore, block annotation is performed based on the concentrated distribution areas of the disease spots.

**Figure 7:**
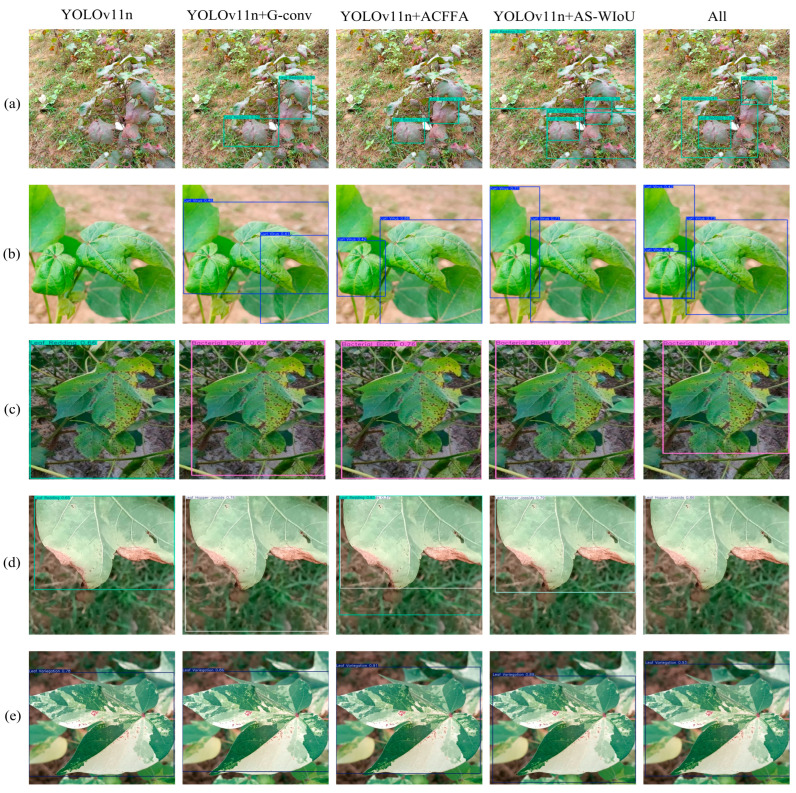
Sample images of the dataset. (**a**) Leaf Redding. (**b**) Curl Virus. (**c**) Bacterial Blight. (**d**) Leaf Hopper Jassids. (**e**) Leaf Variegation.

The final constructed dataset contains a total of six categories: Curl Virus, Healthy, Leaf Hopper Jassids, Leaf Redding, Leaf Variegation, and Bacterial Blight. Examples of each category are shown in Figure 7.

**Figure 8:**
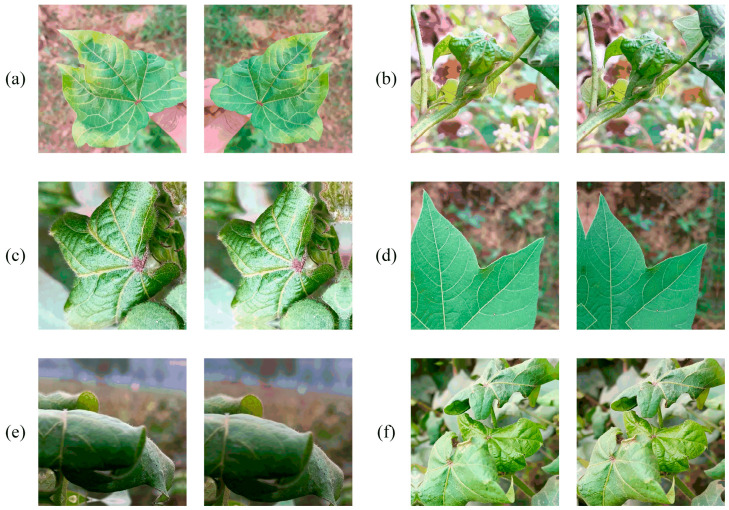
Examples of data augmentation. (**a**) Random flipping. (**b**) Slight rotation. (**c**) Scaling. (**d**) Translation. (**e**) Brightness and contrast adjustment. (**f**) HSV color jitter.

To ensure the independence of model evaluation, the original dataset was stratified by category and divided into training, validation, and test sets at a ratio of approximately 1:1:1. After the division, the original training set contained 406 images, the validation set 406 images, and the test set 412 images. To further improve model generalization and alleviate overfitting, data augmentation operations such as random flipping, slight rotation, scaling, translation, brightness and contrast adjustment, HSV color jitter, Gaussian noise, and mild blurring were applied only to the training set, expanding it to 3248 images. The validation and test sets remained unchanged. This resulted in a dataset containing 4066 images. The distribution details of different diseases across each set are shown in Table 1. Finally, all images were uniformly resampled to 640 × 640 pixels, and labels were saved in YOLO format. Specific examples are shown in Figure 8.

### 3.2. Experimental Environment and Parameter Configuration

To ensure the fairness of experimental comparisons and verify the effectiveness of the improved modules, all experiments are tested under the same hardware environment and parameters. The relevant parameter configurations are listed below.

The experiments used an NVIDIA GeForce RTX 5090 GPU (NVIDIA Corporation, Santa Clara, CA, USA) and an Intel(R) Xeon(R) Gold 649C CPU (Intel Corporation, Santa Clara, CA, USA) for model training and testing, with device memory of 754 GiB. The software environment was built on the Ubuntu 22.04 system (Canonical Ltd., London, UK), using CUDA 12.8 (NVIDIA Corporation, Santa Clara, CA, USA), Python 3.12.3 (Python Software Foundation, Wilmington, DE, USA), and the PyTorch 2.8.0 + cu126 deep learning framework (Linux Foundation, San Francisco, CA, USA) to ensure stable program operation.

Table 2 lists the parameters of the experimental environment. In the experiments, the input image size is uniformly set to 640 × 640 pixels, the batch size is set to 64, SGD is used as the optimizer with a momentum parameter of 0.937 and a weight decay coefficient of 0.0005, and the initial learning rate is 1 × 10^−2^.

As shown in Figure 3, in the improved YOLOv11n backbone network, G-conv is used to replace traditional convolutions to reduce computational cost. The channel expansion ratio is set to s=2, and the convolution kernel size for the linear transformation operation is 5×5. For the four G-conv modules adopted from shallow to deep layers, the target output channel numbers n are respectively set to {32, 64, 128, 256}, and the corresponding intrinsic feature map channel numbers m are {16, 32, 64, 128}.

In the feature fusion stage, the ACFFA module is configured with a 1D convolution kernel size k_size=3, a dilated convolution dilation rate dilation=2, and a channel reduction ratio compress_c=16.

For the AS-WIoU loss function, topk=10 is set to dynamically select the top 10 candidate boxes with the highest prediction scores as positive samples for loss computation, and reg_max=16 is set to discretize the continuous space around the target into 16 sub-intervals. The relevant core parameter configurations are shown in Table 3.

### 3.3. Evaluation Metrics

To objectively evaluate the model performance, precision P is selected to characterize the purity of the prediction results, and recall R reflects the model’s ability to cover real disease spots. The formulas are as follows:

(11) $$P=\frac{TP}{TP+FP},R=\frac{TP}{TP+FN},$$

where TP, FP, and FN represent the numbers of true positive, false positive, and false negative samples, respectively. In view of the inherent antagonistic effect between precision and recall, the F1 score is introduced as the harmonic mean of the two, which is used to comprehensively evaluate the model’s balancing performance between suppressing false positives and avoiding false negatives. The formula is as follows:

(12) $$F1=\frac{2\times P\times R}{P+R}.$$

To comprehensively measure the recognition and localization performance of the model across all categories, this study introduces the mean average precision (*mAP*) as the core evaluation metric of the detector. *mAP* is defined as the area under the precision-recall curve, i.e., the average precision (*AP*). For a single category, the formula is defined as follows:

(13) $$AP=\int_{0}^{1}P(R)dR,$$

where P(R) denotes the precision at a given recall R. *mAP* is the average of *AP* over all categories, and its calculation formula is:

(14) $$mAP=\frac{1}{N}\sum_{i=1}^{N}AP_{i}.$$

The giga floating-point operations (GFLOPs) are introduced to evaluate the resource overhead required for the forward propagation of the algorithm from the perspective of theoretical computational load.

### 3.4. Convergence and Overfitting Analysis

**Figure 9:**
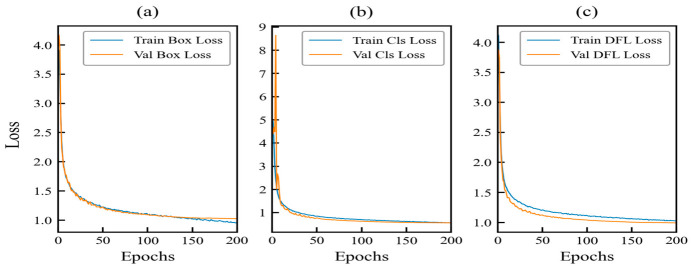
Loss curves of the Ours model during training and validation phases. (**a**) Bounding box regression loss curve. (**b**) Classification loss curve. (**c**) Distribution focal loss curve.

To further evaluate the training stability and generalization ability of the proposed model, the loss curves of the model on the training set and validation set are plotted, as shown in Figure 9.

As shown in Figure 9, the bounding box loss, classification loss, and distribution focal loss all decrease rapidly in the early stage of training, and then gradually level off as the number of epochs increases, indicating that the model can effectively learn the classification information and localization information in the object detection task during training, and gradually reach a convergent state. Among them, the continuous decrease in the bounding box loss and distribution focal loss indicates that the model’s prediction accuracy for target positions and bounding box regression is continuously improved, while the decrease in the classification loss indicates that the model’s discriminative ability for target categories is gradually enhanced. At the same time, the validation loss and training loss are consistent with each other in the overall trend, and the gap between them is small in the later stage, indicating that the model does not show obvious overfitting and has good generalization performance and training stability.

### 3.5. Comparative Experiments on Loss Functions

To verify the performance gain of AS-WIoU in handling cotton foliar spot disease targets, this study conducted a comprehensive comparison with current mainstream loss functions. To ensure the objectivity of the evaluation results, all experiments were executed under the same hardware environment and hyperparameter configurations, all using the improved model architecture built in this study, with only the loss function in the regression branch being replaced to isolate variables. The experimental data are shown in Table 4.

From the global macro performance metrics in Table 4, AS-WIoU outperforms the other six loss functions in recall, *mAP*50, and *mAP*50-95. Specifically, the overall average precision of AS-WIoU across all categories reaches 92.33%, and the overall average recall reaches 87.68%. Meanwhile, *mAP*50 and *mAP*50-95, which measure the core localization accuracy of the detector, reach 90.30% and 73.84%, respectively. Compared with the baseline WIoU-v3 algorithm, our algorithm improves *mAP*50 and *mAP*50-95 by 2.62 and 2.13 percentage points, respectively, demonstrating the unique advantage of the area-sensitive mechanism in handling complex leaf backgrounds and tiny disease spots.

When compared horizontally with the traditional hard intersection-over-union metric CIoU, although the precision of AS-WIoU is slightly lower than that of CIoU (93.76%), its recall reaches 87.68%, the highest in the experimental group, an improvement of 4.4 percentage points over CIoU’s 83.28%. In the task of cotton leaf disease detection, the cost of missed detection (false negatives) is much higher than that of false positives. Missing a single disease spot may lead to disease spread and yield loss, while a small number of false positives may only result in spraying a small amount of preventive pesticide, with relatively limited cost. Therefore, the improvement in recall is more practically significant than a slight decrease in precision. The comprehensive efficiency of AS-WIoU, with an *F*1-score of 89.94%, is also the highest in the experimental group, demonstrating that this loss function has the best robustness in balancing detection accuracy and coverage.

From the microscopic perspective of specific disease categories, AS-WIoU demonstrates strong robustness in handling multi-scale disease spots and low-resolution targets. In the detection tasks of Bacterial Blight and Leaf Variegation, AS-WIoU achieves *mAP*50 of 93.83% and 93.84%, respectively. In contrast, EIoU achieves only 81.16% and 84.78% for these two categories, and Focaler-IoU achieves only 85.76% and 93.26%, showing a clear accuracy advantage of our proposed algorithm. This is mainly attributed to the dynamic physical pixel area monitoring mechanism designed inside AS-WIoU. When the model successfully covers tiny disease spots below a preset threshold, an additional gradient gain is provided through the small target incentive term, effectively alleviating the problems of insufficient gradient contribution of tiny lesions during backpropagation and their susceptibility to information loss in deep feature maps.

Moreover, since all comparative experiments are based on the same backbone network topology, the theoretical computational load and parameter count remain stable at 8.0 GFLOPs and 3.81 M, respectively. It can be seen that the AS-WIoU loss function improves model detection accuracy purely by optimizing the gradient allocation strategy and sample quality evaluation logic during backpropagation, with almost no increase in forward inference cost. In summary, AS-WIoU successfully achieves more accurate and comprehensive bounding box regression of disease spot targets while balancing computational efficiency, verifying its application value in real-time agricultural disease detection scenarios.

### 3.6. Ablation Experiments

To further verify the effectiveness of the improved model in the task of cotton foliar disease detection, this study takes the original YOLOv11n as the baseline model and designs a total of six sets of ablation experiments for lightweight feature extraction, multi-scale feature fusion enhancement, and bounding box regression loss optimization:

1. The original YOLOv11n network.
2. The original YOLOv11n network combined with the G-conv backbone.
3. The original YOLOv11n network combined with the ACFFA module.
4. The original YOLOv11n network combined with the AS-WIoU loss function.
5. The original YOLOv11n network combined with both G-conv and ACFFA.
6. The original YOLOv11n network combined with all proposed improvements, namely the G-conv backbone, the ACFFA module, and the AS-WIoU loss function.

The ablation experiment data are shown in Table 5.

By comparing the original YOLOv11n network with the network incorporating the G-conv backbone, it can be observed that the model achieves an effective gain in computational efficiency. After introducing the lightweight convolution G-conv, the GFLOPs of the model are reduced from 6.3 to 5.6, while its m*AP*50 slightly increases from 88.34% to 88.85%. Although precision, recall, and m*AP*50-95 decrease slightly, the declines are within an acceptable range and can be compensated by subsequent modules. It can be seen that the backbone network with G-conv effectively optimizes the operational efficiency of the backbone by reducing parameter redundancy while maintaining feature representation capability.

The experimental results of the original YOLOv11n network combined with the ACFFA module show that the introduction of the ACFFA module significantly improves the detection performance of the model, with its m*AP*50 increasing substantially from 88.34% of the baseline model to 89.51%, and m*AP*50-95 rising from 71.55% to 73.18%. It can be seen that this module enhances the model’s ability to capture subtle features of cotton leaf disease spots under complex backgrounds through its enhanced multi-scale feature fusion capability and local edge detail repair. Although the increased complexity of the feature aggregation topology raises the computational load from 6.3 GFLOPs to 8.7 GFLOPs, this cost is traded for a more precise cross-scale semantic alignment capability.

After independently introducing the AS-WIoU loss function, the experimental results show that the overall average recall of the model increases from 83.39% of the baseline model to 86.50%, and the m*AP*50 reaches 89.48%. This indicates that the module, through its dynamic adjustment mechanism, can effectively suppress noise interference in farmland environments and improve the refinement of small-target recognition, thereby significantly enhancing the regression stability of the model’s localization branch.

After integrating the G-conv backbone and ACFFA, although the model achieves the highest average precision among all experimental groups, its average recall is only 83.28%. Considering the practical requirements of plant protection and disease control in the field, such a relatively low recall is prone to missed detection of early lesions in practical applications, which may lead to widespread outbreaks of highly infectious diseases. The introduction of the AS-WIoU loss function significantly raises the overall average recall to 87.68%, and the *mAP*50-95 further increases from 71.68% to 73.84%. This performance bias towards recall effectively reduces the missed detection rate of highly infectious diseases, which meets the actual needs of agricultural scenarios.

The final improved scheme achieves an overall average precision of 92.33%, an average recall of 87.68%, *mAP*50 of 90.30%, and *mAP*50-95 of 73.84%, reaching the best comprehensive performance among all experimental groups. Although the theoretical computational load under the synergistic effect of multiple modules increases from 6.3 GFLOPs of the baseline model to 8.0 GFLOPs, it still effectively meets the engineering requirements of real-time monitoring. Moreover, a significant enhancement in generalization ability is achieved in subsequent generalization experiments, demonstrating that the model achieves a balance between practicality and operational efficiency in complex agricultural environments.

**Figure 10:**
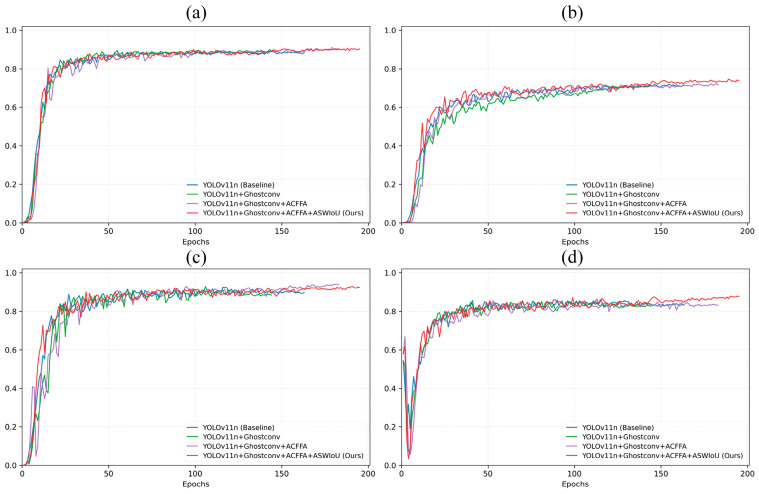
Training curves of the ablation study. (**a**) mAP@50. (**b**) mAP@50-95. (**c**) Precision. (**d**) Recall.

As shown in Figure 10, to visually verify the impact of each improved module on the learning ability of the model, the evolution curves of *mAP*50, *mAP*50-95, precision, and recall over 200 epochs in the ablation experiments are recorded.

In terms of convergence speed and stability, all models exhibit extremely high convergence efficiency during the first 50 epochs, with various metrics rising rapidly. The model with G-conv, despite a significant reduction in parameter count, shows a convergence trajectory that closely aligns with the baseline model and stabilizes in later stages, confirming that the lightweight modification preserves feature extraction capability while ensuring robust training. From the perspective of cumulative performance gains, as the ACFFA module and AS-WIoU loss function are integrated sequentially, the orange curve representing the final improved model gradually moves above the baseline model envelope in the mid-to-late training stages. Specifically on *mAP*50-95 and recall, the final improved model consistently ranks highest in the overall trend after a certain number of epochs, demonstrating clear performance superiority. It can be seen that ACFFA’s adaptive fusion of cross-scale features and AS-WIoU’s precise correction of lesion localization effectively raise the upper bound for capturing complex morphologies of cotton diseases.

**Figure 11:**
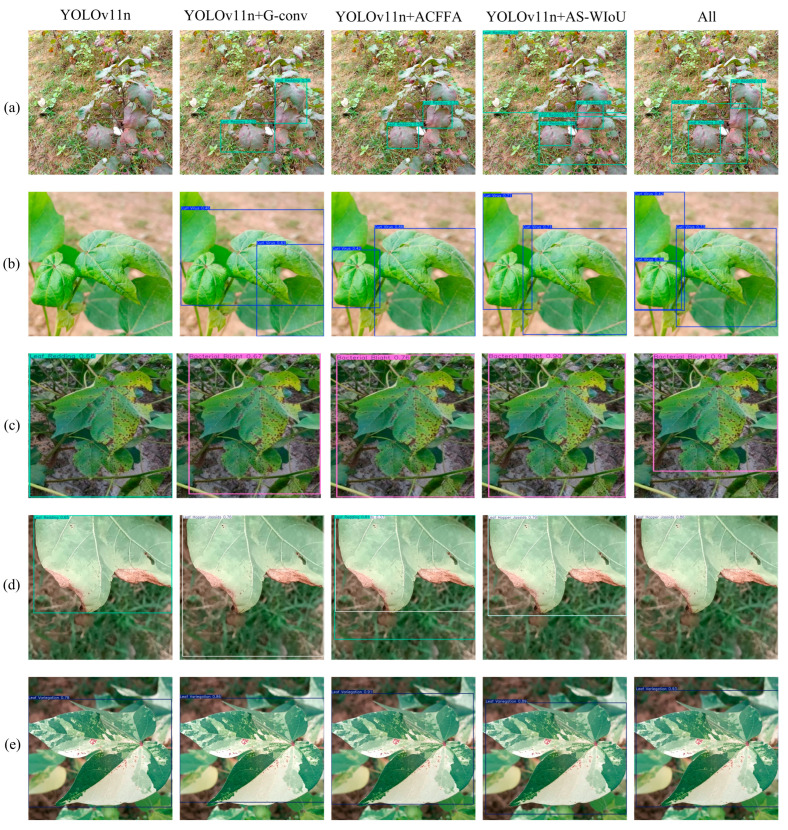
Visualization results of the experiments. (**a**) Leaf Redding, (**b**) Curl Virus, (**c**) Bacterial Blight, (**d**) Leaf Hopper Jassids, (**e**) Leaf Variegation.

To more intuitively illustrate the contribution of each improved component, several typical images are selected for comparison, and the comparison results are shown in Figure 11.

As shown in Figure 11, the visualization results of the ablation experiments demonstrate the detection performance of the model on five types of cotton diseases (Leaf Redding, Curl Virus, Bacterial Blight, Leaf Hopper Jassids, and Leaf Variegation) after the introduction of each improved module. Different diseases exhibit significant differences in target scale, lesion morphology, background complexity, and imaging quality, thus comprehensively reflecting the effectiveness of each module in real field scenarios.

As shown in the first row corresponding to the Leaf Redding scenario. This type of disease region is mainly distributed in the upper parts of the plant and local leaf areas, with relatively small lesion scales, and is subject to interference from complex backgrounds such as soil, weeds, and branches and leaves, representing a disease detection scenario under low-quality image conditions. The baseline YOLOv11n has insufficient response to small-scale, weak-textured disease spots, and is prone to missed detections and detection box offsets. After introducing G-conv, the model enhances basic feature extraction capability while maintaining lightweight design, making the feature response in small-target regions more stable. With the addition of ACFFA, the capabilities of cross-layer feature alignment and local detail compensation are improved, enabling the model to better perceive scattered disease spots and their edge information. After incorporating AS-WIoU, the bounding box regression process imposes more reasonable constraints on small targets and low-quality candidate boxes, allowing the complete model to cover multiple disease regions, indicating that the proposed method has good adaptability for small-target disease spot detection under complex field backgrounds.

The second row corresponds to the Curl Virus scenario. This type of disease manifests as leaf curling, yellowing, and deformation, with the disease region having a large spatial span but irregular boundary morphology, and the texture of the disease spots shares some similarity with healthy leaf regions. ACFFA achieves cross-layer feature alignment through channel prior calibration and compensates for detail information using dilated convolution, helping to alleviate the spatial information loss caused by downsampling. Therefore, after introducing ACFFA, the model’s perception capability for curled leaf edges and irregular disease regions is enhanced, and the matching between detection boxes and disease regions becomes more sufficient.

The third row corresponds to the Bacterial Blight scenario. This type of disease typically presents as scattered small spots and local necrotic areas on the leaf surface, which can easily be confused with leaf veins, shadows, and surrounding branches and leaves. The baseline model has insufficient response to fine-grained disease spot textures, resulting in a loose match between detection boxes and actual lesion regions. After introducing G-conv, the model extracts basic texture features more adequately. ACFFA alleviates multi-scale semantic conflicts, enabling better fusion of high-level semantic information with shallow spatial details. AS-WIoU optimizes the localization process through adaptive sample quality assessment. The complete model can better cover dense disease spot regions while maintaining stable localization of the overall leaf area, indicating that the improved feature fusion and localization constraints help enhance the model’s perception of fragmented disease spots and irregular boundaries.

The fourth row corresponds to the Leaf Hopper Jassids scenario. This sample suffers from issues such as uneven illumination and blurred leaf edges. Due to the weak visual distinction between the disease region and naturally aged leaf areas or shadow regions, the baseline model is prone to unreasonable detection box coverage or boundary drift. AS-WIoU dynamically evaluates sample quality through a target area threshold filtering mechanism and a blur-aware weight factor, enabling the model to adjust localization constraints according to image quality and target characteristics, thereby reducing the impact of low-quality annotation noise and blurred boundaries on the regression process. Compared with the baseline model, the detection boxes after incorporating AS-WIoU are more consistent with the damaged leaf areas, indicating that this loss function can improve localization robustness under complex backgrounds and low-quality samples.

The fifth row corresponds to the Leaf Variegation scenario. This type of disease manifests as large-scale mottled and chlorotic areas on the leaf surface, with relatively large target scales, but the disease texture is easily confused with strong light reflection, leaf overlap, and background soil. Unlike the small-target disease spots in the first row, this scenario mainly examines the model’s ability to completely cover large-scale, diffuse disease regions. The complete model maintains a relatively stable detection box range for this type of sample, indicating that the proposed method not only improves detection of small targets and fine-grained disease spots, but also adapts to the localization requirements of large-scale disease regions.

Overall, Figure 11 validates the role of each module from different disease types. G-conv mainly replaces some standard convolutions with Ghost convolutions, maintaining the basic feature representation capability of the lightweight backbone while reducing parameter overhead. ACFFA enhances the model’s perception of multi-scale disease spots, leaf edges, and irregular lesion regions through channel prior calibration, cross-layer feature alignment, and dilated convolution detail compensation. AS-WIoU further improves bounding box regression performance for small targets, low-quality images, and samples with blurred boundaries through adaptive sample quality evaluation and blur-aware localization constraints. From the comprehensive visualization results, it can be seen that the first and fifth rows illustrate the detection differences between small-scale disease spots and large-scale disease regions, the third row highlights the need for multi-scale fine-grained disease spot recognition, and the fourth row reflects the localization challenges under low-quality images and complex backgrounds. The complete model demonstrates higher detection completeness and localization stability in all the above scenarios, indicating that G-conv, ACFFA, and AS-WIoU synergistically improve the robustness and adaptability of the model in real-world cotton disease detection tasks from three aspects: feature extraction, multi-scale information fusion, and bounding box optimization.

**Figure 12:**
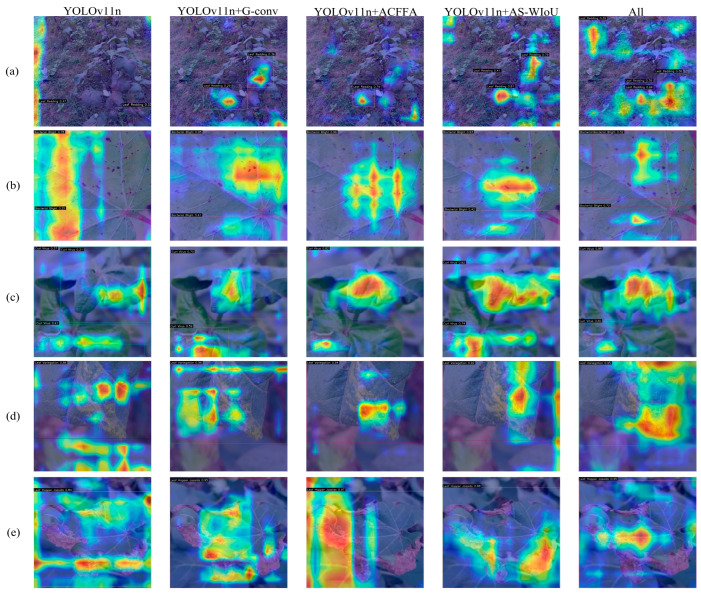
Heatmap of the experiment. (**a**) Leaf Redding, (**b**) Bacterial Blight, (**c**) Curl Virus, (**d**) Leaf Variegation, (**e**) Leaf Hopper Jassids.

To further explore the feature extraction mechanism and decision-making basis of the model during the detection process, a comparative analysis of the heatmaps generated by the improved models at each stage is conducted, with one representative disease spot selected from each category, as shown in Figure 12.

The original YOLOv11n, due to insufficient feature interaction capability between different levels, fails to fully cover disease spots with its heatmap response regions when facing complex disease spots. Specifically, in the first row (Leaf Redding) and the fourth row (Leaf Variegation), the response regions of the original model are scattered for small-target disease spots and diffuse mottled areas, and are easily disturbed by leaf texture and background. For the multi-scale and irregular disease spots in the second row (Bacterial Blight) and the third row (Curl Virus), the heatmap responses exhibit a fragmented distribution, indicating insufficient overall disease spot perception capability. In the fifth row (Leaf Hopper Jassids), the model’s activation for tiny disease spots and edge features remains inadequate.

After introducing G-conv, the model maintains a lightweight design while focusing more robustly on the main disease spot regions, with significantly enhanced activation for small targets and low-contrast disease spots, indicating improved feature extraction capability. For example, in the first row (Leaf Redding), G-conv causes the heatmap response, which was originally scattered in background areas, to gradually concentrate on local small disease spots. In the fifth row (Leaf Hopper Jassids), the activation intensity for tiny disease spots and leaf edge regions is also increased. After integrating the ACFFA module into the original model, the model’s perception capability for multi-scale disease spots and their edge information is enhanced, with the heatmap response gradually changing from discrete points to continuous blocks, covering the disease spot regions more completely. For instance, in the second row (Bacterial Blight), the activation in dense small spot areas is significantly increased. In the third row (Curl Virus), curled leaves and their irregular edge regions receive more continuous response coverage, indicating that ACFFA effectively alleviates multi-scale semantic conflicts and edge information loss. After combining the original model with the AS-WIoU loss function, the model’s boundaries become tighter, the heatmap response concentrates on the core areas of disease spots, background interference is significantly reduced, and boundary localization becomes clearer. For example, in the fourth row (Leaf Variegation), the heatmap response gradually converges from a large, scattered activation to the mottled lesion areas. In the Curl Virus and Leaf Hopper Jassids samples, the response in non-disease background regions decreases, and the core areas of disease spots become more prominent. The complete model exhibits more accurate and continuous disease spot attention capability across all disease scenarios, verifying the effectiveness of G-conv, ACFFA, and AS-WIoU in feature representation, multi-scale information fusion, and boundary optimization.

### 3.7. Comparative Experiment

To further validate the competitive advantage of the above model in the task of cotton disease recognition, this study conducted a horizontal evaluation of the improved YOLOv11 model against a variety of current mainstream object detection algorithms. Table 6 records the performance of each model on core metrics such as precision, recall, *F*1-score, mean average precision, and parameter count, providing empirical support for the engineering deployment of this system in agricultural monitoring equipment.

Based on the comparative experimental results in Table 6, the improved model proposed in this study shows significant advantages in detection accuracy. Its precision, recall, *F*1-score, *mAP*50, and *mAP*50-95 reach 92.33%, 87.68%,89.94%, 90.30%, and 73.84%, respectively, which are the best among the compared models. Compared with the original YOLOv11n model, the improved model achieves improvements of 2.73, 4.29, 3.56, 1.96, and 2.29 percentage points in precision, recall, *F*1-score, *mAP*50, and *mAP*50-95, respectively, indicating that the proposed improvement strategy effectively enhances the model’s recognition capability and localization accuracy for cotton disease targets. Particularly on the stricter comprehensive evaluation metric *mAP*50-95, the improved model achieves the highest value, demonstrating that it not only accurately identifies disease categories but also maintains good bounding box localization stability under different IoU thresholds, making it more suitable for detection scenarios with significant variations in disease spot scales and irregular edge features under complex field backgrounds.

Compared with other lightweight YOLO series models, the proposed model achieves higher detection performance while maintaining lower complexity. For example, although YOLOv9t has only 1.97 M parameters, its *mAP*50 and *mAP*50-95 are 88.38% and 71.80%, respectively, both lower than those of the proposed model. YOLOv8n achieves an *mAP*50-95 of 70.86%, which is 2.98 percentage points lower than the proposed model. This indicates that simply reducing the parameter count does not guarantee comprehensive performance in disease detection tasks, while the proposed improved model can further enhance feature representation capability for small-scale disease spots and targets under complex backgrounds within an acceptable computational cost range.

It is worth noting that YOLOv8n achieves relatively prominent detection accuracy in Table 6, even outperforming some newer versions of the YOLO model. This observation suggests that it is necessary to evaluate the impact of training stochasticity on performance assessment from a statistical perspective. To assess the influence of training randomness on performance evaluation, this study selects YOLOv8n, YOLOv11n, and the proposed improved model to conduct five independent repeated training runs (with random seeds 0, 1, 2, 3, 4). Each run is trained from scratch to cover different weight initializations, data augmentation sequences, and batch shuffling orders, while a fixed seed is uniformly used during validation to ensure consistency in the inference process. The selection of these three models is based on the following considerations: YOLOv8n performs prominently in a single validation run, and its stability needs to be confirmed through multiple random seeds; YOLOv11n serves as the direct baseline for our improvements and provides a reference benchmark for measuring the stability of the improvement gains; and the improved model is the core subject for verifying the effectiveness of the proposed improvement strategy.

The experimental results show that YOLOv8n achieves an *mAP*50 of 89.24% ± 0.81%, an *mAP*50-95 of 70.83% ± 0.57%, a Precision of 90.54% ± 1.02%, and a Recall of 84.29% ± 0.94% over the five independent runs. The single-run result (89.16%) falls within this fluctuation range, indicating that the original single-run result does not significantly deviate from its average performance, but certain stochastic fluctuation does exist. YOLOv11n achieves an *mAP*50 of 88.62% ± 0.54%, an *mAP*50-95 of 71.86% ± 0.44%, a Precision of 89.15% ± 0.95%, and a Recall of 84.35% ± 1.07% over the five independent runs. Our improved model achieves an *mAP*50 of 90.14% ± 0.73%, an *mAP*50-95 of 73.89% ± 0.46%, a Precision of 91.75% ± 0.94%, and a Recall of 88.07% ± 0.72% over the five independent runs. The standard deviations of all metrics for the three models are relatively small (with the vast majority of relative proportions within 1%), indicating that the training processes of each model are highly reproducible.

On the *mAP*50 metric, the proposed improved model consistently outperforms both YOLOv11n and YOLOv8n across all five independent runs, indicating that the performance gains brought by the improvements are not a product of stochastic fluctuation but are statistically stable. Meanwhile, the prominent performance of YOLOv8n is indeed partially attributable to its architectural characteristics; however, multiple experiments confirm that the average gap of 0.90 percentage points between it and our improved model is statistically stable.

Compared with existing state-of-the-art cotton disease detection algorithms, the proposed model achieves a better trade-off between accuracy and lightweight design. In the performance evaluation, compared with RDL-YOLO, the proposed model significantly reduces the computational cost from 10.4 GFLOPs to 8.0 GFLOPs, while achieving a counter-trend increase of 0.68 percentage points in *mAP*50-95. Furthermore, when compared with the Improved YOLOv8s model, the proposed method not only substantially reduces the parameter count by approximately 53% (from 8.1 M to 3.81 M) and the computational cost by about 65% (from 23.1 GFLOPs to 8.0 GFLOPs), but also achieves an improvement of 0.97 percentage points in *mAP*50-95. The above comparative results fully demonstrate that the improved network architecture effectively breaks through the accuracy bottleneck of multi-scale disease detection while alleviating the deployment burden on agricultural edge devices to a certain extent.

It should be noted that the parameter counts and computational costs of the compared models in Table 6 vary to some extent. Therefore, when conducting horizontal comparisons, it is necessary to clarify the fairness of cross-budget comparisons. Our model (3.81 M, 8.0 GFLOPs) lies between lightweight models (e.g., YOLOv9t, 1.97 M/7.6 GFLOPs) and large-scale models (e.g., RT-DETR-L, 32.0 M/103.5 GFLOPs). This comparison is not intended to directly rank models of different sizes on the same axis, but rather to provide readers with a unified evaluation benchmark for examining the accuracy–efficiency trade-offs of different architectures in the cotton disease detection task. Experimental results show that compared with RT-DETR-L (69.16%), our model achieves a higher *mAP*50-95 of 73.84% with only 7.7% of the computational cost. Meanwhile, compared with YOLOv9t and YOLOv11n, our model shows a slight increase in parameter count and computational cost, but improves *mAP*50-95 by 2.04 and 2.29 percentage points, respectively. This trade-off is reasonable in agricultural embedded scenarios: a moderate increase in model capacity within the allowable computational budget can effectively enhance detection robustness for small-scale lesions and complex backgrounds. Therefore, despite the varying model scales, our comparison still provides meaningful references for model selection under different resource constraints.

In summary, the improved model achieves favorable overall performance in cotton disease detection, enabling accurate localization of small-scale disease spots while retaining a certain degree of real-time processing capability, thereby verifying the feasibility and advancement of deploying this model in embedded field monitoring equipment.

### 3.8. Cross-Host Generalization Experiment

To further verify the generalization ability and universality of the improved model across different crop disease detection tasks, this study selects three representative crop disease datasets from the Multi-Crop Disease Dataset [51] on the Mendeley Data platform, namely banana leaf spot, groundnut leaf spot, and radish leaf spot, to conduct cross-host generalization experiments. The proposed model is compared with current mainstream models and state-of-the-art peer models. The experiments use the same experimental environment and configuration parameters as described in Section 3.2. For each of the above three crop datasets, the data are re-split into training, validation, and test sets at a ratio of 7:2:1, and the model is retrained from scratch to evaluate its performance on different crop tasks. The detailed sample composition of each dataset is shown in Table 7, and the experimental results are presented in Table 8.

The cross-host generalization experimental results are shown in Table 8. It should be noted that different crop disease datasets exhibit objective visual differences and domain shifts in terms of image acquisition conditions, disease phenotypic characteristics, and field background complexity. Consequently, the actual performance of each model on different transfer tasks shows certain fluctuations and divergences, which are common in cross-crop transfer scenarios. Specifically, on the banana disease dataset, our model achieves an *mAP*50 of 73.55%, slightly lower than RDL-YOLO (75.92%) and YOLOv12n (74.67%), but its *mAP*50-95 (56.55%) remains within 0.33 percentage points of the best value (56.88%). In terms of computational cost, our model (8.0 GFLOPs, 3.82 M) exhibits better lightweight characteristics compared with RDL-YOLO (10.4 GFLOPs, 3.54 M) and Improved YOLO (23.2 GFLOPs, 8.13 M). On the groundnut dataset, our model achieves a Precision of 92.71%, the highest among the compared models. On the radish dataset, our model attains an *mAP*50 of 88.96%, also achieving competitive detection performance. Considering the overall performance across the three cross-host datasets, our architecture achieves competitive results on the groundnut and radish disease detection tasks while maintaining a reasonable computational cost, and demonstrates a lightweight trade-off comparable to the best methods on the banana disease detection task. These results indicate that the proposed architecture exhibits certain transfer potential across different crop disease detection tasks, but its generalization performance is considerably influenced by the image acquisition conditions and disease phenotypic characteristics of the target dataset, and thus still requires adaptation and optimization for specific crops in practical deployment.

### 3.9. Edge Deployment Testing

**Figure 13:**
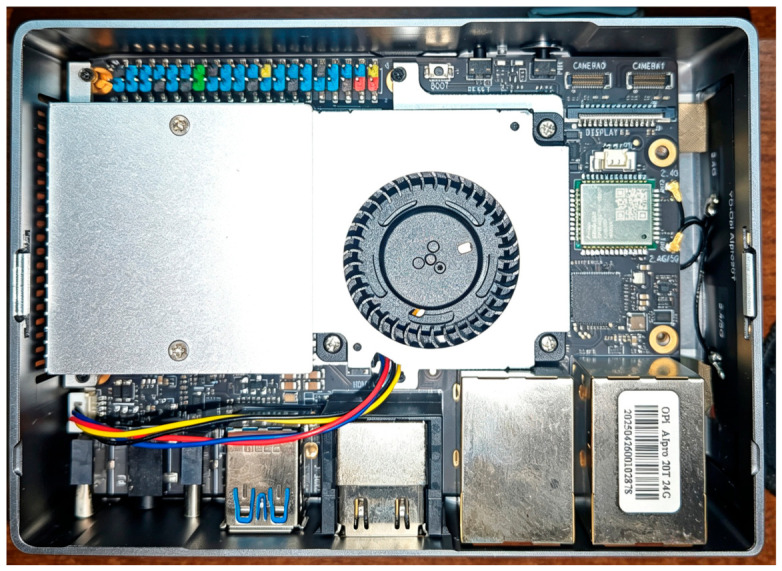
Physical board of the OrangePi AIpro (20T).

To further verify the deployment feasibility and real-time inference capability of the proposed model in real agricultural embedded scenarios, this study selects the OrangePi AIpro (20T) development board (Shenzhen Xunlong Software Co., Ltd., Shenzhen, China) as the edge-side test platform. The board is equipped with a quad-core 64-bit processor and an AI processor, delivering 20 TOPS of AI computing power (INT8), along with 12 GB LPDDR4X memory. It supports the Ubuntu operating system and combines high computing power with low power consumption, making it widely applicable to AI edge computing and smart agriculture scenarios. The physical board of the OrangePi AIpro (20T) is shown in Figure 13.

Model deployment adopts the Huawei Ascend CANN inference backend. The deployment process is as follows: first, the trained PyTorch weight file (.pt) is exported to ONNX format. During the export process, the model input size is fixed to 640 × 640, the opset version is set to 11, and NMS post-processing is disabled, retaining only the forward inference part of the model. Subsequently, the Huawei Ascend ATC tool is used to convert the ONNX model to the OM offline model format compatible with the Ascend NPU. During the conversion, the --soc_version parameter is set according to the target device NPU model, and --input_shape is fixed to images:13,640,640. Model deployment adopts FP16 precision to reduce inference computational overhead. OM model loading and inference are implemented based on the AscendCL interface under the CANN environment. Since the NMS operator is not encapsulated into the ONNX model in the current deployment pipeline, the model performs only forward inference on the NPU, while post-processing operations such as confidence filtering, bounding box restoration, and NMS are completed on the CPU side.

During the testing phase, images from the validation set that were not involved in training are selected for deployment performance validation. The input images are resized and padded to 640 × 640 pixels using the letterbox method with aspect ratio preservation. To evaluate the inference performance of the model on the edge-side NPU, the average latency of the forward inference stage is measured. Each image is inferred 100 times after a warm-up phase, and the average value is taken as the final result.

Experimental results show that the proposed model achieves an inference speed of 31.59 FPS on the NPU of the OrangePi AIpro (20T), which can meet the real-time detection requirements of field video streams. This result confirms that, while maintaining detection accuracy advantages, the proposed model can achieve basic real-time inference on 20 TOPS-class edge computing platforms, demonstrating a certain degree of engineering feasibility for deployment on agricultural embedded terminals. Future work can further improve the actual runtime efficiency on edge devices through techniques such as model quantization, operator fusion, and inference pipeline optimization.

## 4. Discussion

To address the challenges in cotton foliar lesion detection, including computational redundancy, varying target scales, complex background noise interference, and limited detection accuracy for early-stage disease spots, an improved detection model based on YOLOv11n is proposed. By introducing the G-conv backbone network, the model maintains robust feature extraction capability while reducing computational load. The integration of the ACFFA module further optimizes weight allocation in the feature fusion process, enhancing the system’s perceptual depth for multi-scale lesions. The AS-WIoU loss function, through the synergy of a clarity-aware mechanism and an area-sensitive mechanism, achieves effective correction of disease spot localization.

Experimental results show that on the established cotton disease dataset, the proposed model achieves a precision and recall of 92.33% and 87.68%, respectively, and *mAP*50 and *mAP*50-95 reach 90.30% and 73.84%, which are improvements of 1.96% and 2.29% over the original YOLOv11n, respectively. It can effectively cope with the detection pressure caused by complex field environments. Although the introduction of the enhanced modules leads to a slight increase in computational cost, the parameter count of 3.81 M still meets the performance requirements of embedded detection devices in real-time operations. It can be seen that the proposed model achieves an effective fusion of computational efficiency, detection accuracy, and localization robustness, demonstrating good practical application performance.

At the same time, it should be acknowledged that the annotation strategy may introduce certain effects on the detection results. To accommodate the phenotypic differences among various diseases, this study adopts a differentiated annotation strategy (see Section 3.1), i.e., using whole-leaf boxes for large-scale systemic diseases, discrete boxes for isolated lesions, and regional blocks for dense spots. While this strategy improves the model’s adaptability to complex field diseases, it also introduces some side effects that deserve attention. First, the coexistence of annotation boxes at different scales may cause optimization bias during training: whole-leaf boxes often occupy 30–70% of the image area, whereas local lesion boxes may cover only 0.5–2%. IoU is more tolerant to positional shifts for large-scale targets but more sensitive for small-scale ones. Although AS-WIoU includes a small-target incentive term to enhance the gradient contribution of tiny lesions, the inherent scale disparity may still result in lower localization accuracy for small lesions compared with large-box targets. Second, when the same leaf contains both large- and small-box annotations of the same disease category with similar confidence scores, NMS may incorrectly suppress one of them. Furthermore, due to their larger scales, whole-leaf boxes are more likely to achieve higher AP values under the IoU evaluation framework, which may artificially inflate the overall mAP and cause a deviation between the evaluation results and the practical requirements of quantitatively assessing lesion count, distribution, and severity grading in real applications.

In terms of model architecture, although the current model performs robustly in conventional field scenarios, its generalization ability in extreme agricultural environments, such as intense lighting, high humidity, and severe occlusion, still remains to be verified. Meanwhile, on micro embedded chips with extremely limited computational resources, the additional computational overhead introduced by the ACFFA module still needs further reduction to meet more stringent real-time requirements.

To address the above limitations, future research will be advanced synergistically from both the data and model perspectives. At the model level, lightweight techniques such as knowledge distillation and operator fusion will be explored to improve the operational efficiency and generalization ability of the model without significantly increasing the parameter burden. At the task level, methods such as semantic segmentation, density estimation, or multi-task learning will be considered to further enable quantitative assessment of disease spots beyond object detection, so as to better meet the practical application requirements of disease grading and precise prevention and control.

## 5. Conclusions

This study proposes an optimized detection framework aimed at addressing two key bottlenecks in cotton disease monitoring: computational redundancy and multi-scale lesion variation. By integrating the G-conv backbone, the ACFFA module, and the AS-WIoU loss function, the final model achieves a delicate balance between detection accuracy and structural efficiency. Empirical validation on the established cotton disease dataset shows that the proposed architecture improves *mAP*50 by 1.96% and *mAP*50-95 by 2.29% over the original YOLOv11n, while maintaining a parameter count of 3.81 M.

## Figures and Tables

**Table 1:** Detailed distribution statistics of the cotton leaf disease dataset.

Class	Train	Val	Test	Objects
Curl Virus	904	108	109	1121
Healthy	560	67	72	699
Leaf Hopper Jassids	432	52	54	538
Leaf Redding	1200	150	140	1490
Leaf Variegation	312	38	40	390
Bacterial Blight	296	36	38	370
Number of objects	3704	451	453	4608
Number of images	3248	406	412	4066

**Table 2:** Experimental environment statistics.

Hardware and Software Environment	Configuration
GPU	NVIDIA GeForce RTX 5090
CPU	Intel(R) Xeon(R) Gold 649C
Python	3.12.3
Pytorch	2.8.0 + cu128
CUDA	12.8

**Table 3:** Core parameter configurations of the Adaptive Scale-aware Wise Intersection over Union (AS-WIoU) loss function.

Symbol	Value	Meaning
$w_{soft}$	0.7	soft weight coefficient
α	1.9	steepness of non-monotonic focusing curve
δ	3.0	peak points of gradient gain for r
$\tau_{blur}$	0.3	blur threshold
$A_{min}$	32.0	small-area threshold
$\omega_{reward}$	0.01	incentive coefficient for small targets

**Table 4:** Comparative experimental results of different loss functions.

Loss Function	Type	Precision (%)	Recall (%)	*F*1-Score	*mAP*50 (%)	*mAP*50-95 (%)	GFLOPs	Params (M)
CIoU	Curl Virus	95.10	79.63	86.68	93.09	67.53	8.0	3.81
	Healthy	96.69	81.62	88.53	88.27	71.44		
	Leaf Hopper Jassids	87.28	94.92	90.95	85.07	65.84		
	Leaf Redding	88.50	84.00	86.19	90.02	73.32		
	Leaf Variegation	95.02	91.21	93.08	93.99	75.27		
	Bacterial Blight	100.0	80.56	89.23	89.77	76.68		
	all	93.76	83.28	88.21	90.03	71.68		
DIoU	Curl Virus	94.37	83.33	88.51	91.76	70.07	8.0	3.81
	Healthy	85.49	79.16	82.21	85.04	70.38		
	Leaf Hopper Jassids	78.48	90.38	84.01	82.15	67.92		
	Leaf Redding	93.49	86.21	89.70	91.87	74.13		
	Leaf Variegation	100.0	90.11	94.80	94.07	75.04		
	Bacterial Blight	93.19	75.99	83.71	88.62	75.19		
	all	90.84	84.20	87.16	88.92	72.12		
EIoU	Curl Virus	89.83	81.83	85.64	88.40	65.77	8.0	3.81
	Healthy	93.00	79.37	85.65	87.93	74.84		
	Leaf Hopper Jassids	82.70	84.62	83.65	86.66	68.15		
	Leaf Redding	84.46	85.33	84.90	79.58	70.43		
	Leaf Variegation	92.66	89.47	91.04	84.78	75.52		
	Bacterial Blight	93.26	80.56	86.44	81.16	74.41		
	all	89.32	83.53	86.22	79.58	71.52		
WIoU-v1	Curl Virus	91.95	80.56	85.88	89.99	68.39	8.0	3.81
	Healthy	90.91	79.10	84.60	87.27	74.96		
	Leaf Hopper Jassids	81.86	84.62	83.21	81.99	65.73		
	Leaf Redding	92.65	88.67	90.62	92.29	72.44		
	Leaf Variegation	95.69	86.84	91.05	90.74	79.46		
	Bacterial Blight	95.07	77.78	85.56	90.79	75.61		
	all	91.36	82.93	86.82	88.84	72.77		
WIoU-v3	Curl Virus	96.60	81.48	88.40	92.73	68.56	8.0	3.81
	Healthy	79.95	83.33	81.60	86.56	72.41		
	Leaf Hopper Jassids	69.26	88.46	77.69	80.87	65.80		
	Leaf Redding	90.13	85.27	87.64	92.91	73.09		
	Leaf Variegation	94.59	86.84	90.55	91.00	79.17		
	Bacterial Blight	82.03	76.10	78.95	81.99	71.24		
	all	85.43	83.58	84.14	87.68	71.71		
Focaler-IoU	Curl Virus	92.27	77.34	84.15	87.84	64.27	8.0	3.81
	Healthy	90.10	79.10	84.24	85.63	70.84		
	Leaf Hopper Jassids	84.46	83.64	84.05	84.91	68.11		
	Leaf Redding	90.78	88.00	89.37	91.75	70.86		
	Leaf Variegation	99.19	89.47	94.08	93.26	78.78		
	Bacterial Blight	93.17	80.56	86.40	85.76	71.39		
	all	91.66	83.02	87.05	88.19	70.71		
AS-WIoU	Curl Virus	96.99	87.04	91.75	91.75	69.42	8.0	3.81
	Healthy	90.11	82.09	87.06	87.06	75.38		
	Leaf Hopper Jassids	84.92	88.46	84.18	84.18	68.45		
	Leaf Redding	89.57	85.45	91.11	91.11	72.74		
	Leaf Variegation	97.20	91.36	93.84	93.84	80.44		
	Bacterial Blight	95.18	91.67	93.83	93.83	76.59		
	all	92.33	87.68	89.94	90.30	73.84		

**Table 5:** Ablation experimental results of each module.

Network	Type	Precision (%)	Recall (%)	*F*1-Score	*mAP*50 (%)	*mAP*50-95 (%)	GFLOPs	Params (M)
YOLOv11n	Curl Virus	95.63	82.41	88.53	90.30	68.54	6.3	2.58
	Healthy	87.20	77.35	81.98	85.23	71.90		
	Leaf Hopper Jassids	80.23	84.62	82.37	84.84	63.94		
	Leaf Redding	97.25	83.33	89.75	86.56	68.32		
	Leaf Variegation	97.21	92.11	94.59	94.16	90.01		
	Bacterial Blight	90.07	80.56	85.05	88.93	76.61		
	all	89.60	83.39	86.38	88.34	71.55		
YOLOv11n + G-conv Backbone	Curl Virus	93.44	80.56	86.52	91.44	64.44	5.6	2.35
	Healthy	88.99	74.63	81.18	88.14	72.17		
	Leaf Hopper Jassids	80.37	84.62	82.44	85.04	67.56		
	Leaf Redding	82.77	87.23	84.94	82.84	66.56		
	Leaf Variegation	97.20	92.11	94.59	93.92	78.82		
	Bacterial Blight	87.22	76.29	81.39	91.75	76.18		
	all	88.33	82.57	85.35	88.85	70.96		
YOLOv11n + ACFFA	Curl Virus	98.64	81.48	89.24	91.21	67.55	8.7	4.04
	Healthy	92.77	79.10	85.39	87.27	73.27		
	Leaf Hopper Jassids	82.25	86.54	84.34	86.90	70.37		
	Leaf Redding	86.31	84.06	85.17	87.30	72.98		
	Leaf Variegation	90.31	92.11	91.20	84.01	81.63		
	Bacterial Blight	89.56	83.33	86.33	90.37	73.32		
	all	89.97	84.44	87.12	89.51	73.18		
YOLOv11n + AS-WIoU	Curl Virus	93.04	86.11	90.22	90.22	65.47	6.3	2.58
	Healthy	81.61	84.46	87.42	87.42	71.65		
	Leaf Hopper Jassids	79.03	86.54	87.32	87.32	67.58		
	Leaf Redding	88.29	80.00	88.07	88.07	68.11		
	Leaf Variegation	89.67	91.31	93.88	93.88	75.24		
	Bacterial Blight	96.68	80.56	90.00	90.00	73.47		
	all	88.05	86.50	89.48	89.48	70.25		
YOLOv11n + G-conv Backbone+ ACFFA	Curl Virus	95.10	79.63	86.68	93.09	67.53	8.0	3.81
	Healthy	96.69	81.62	88.53	88.27	71.44		
	Leaf Hopper Jassids	87.28	94.92	90.95	85.07	65.84		
	Leaf Redding	88.50	84.00	86.19	90.02	73.32		
	Leaf Variegation	95.02	91.21	93.08	93.99	75.27		
	Bacterial Blight	100.0	80.56	89.23	89.77	76.68		
	all	93.76	83.28	88.21	90.03	71.68		
Ours	Curl Virus	96.99	87.04	91.75	91.75	69.42	8.0	3.81
	Healthy	90.11	82.09	87.06	87.06	75.38		
	Leaf Hopper Jassids	84.92	88.46	84.18	84.18	68.45		
	Leaf Redding	89.57	85.45	91.11	91.11	72.74		
	Leaf Variegation	97.20	91.36	93.84	93.84	80.44		
	Bacterial Blight	95.18	91.67	93.83	93.83	76.59		
	all	92.33	87.68	89.94	90.30	73.84		

**Table 6:** Comparative experimental results of different methods.

Method	Precision (%)	Recall (%)	*F*1-Score	*mAP*50 (%)	*mAP* 50-95 (%)	GFLOPs	Params (M)
YOLOv26n [45]	89.94	82.16	85.88	87.80	68.03	5.6	2.41
YOLOv13n [46]	88.51	82.40	85.36	87.83	70.47	6.2	2.47
YOLOv12n [47]	83.25	79.21	81.19	83.97	60.38	6.3	2.56
YOLOv11n [30]	89.60	83.39	86.38	88.34	71.55	6.3	2.58
YOLOv10n [48]	87.02	80.47	83.62	86.99	68.74	6.5	2.27
YOLOv9t [49]	87.62	81.87	84.66	88.38	71.80	7.6	1.97
YOLOv8n [50]	91.37	85.01	88.09	89.16	70.86	8.1	3.00
RT-DETR-L [21]	86.67	83.75	85.21	85.41	69.16	103.5	32.00
RDL-YOLO [40]	88.94	87.06	87.99	88.95	73.16	10.4	3.54
Improved YOLOv8s [17]	90.41	85.79	88.06	89.29	72.87	23.1	8.1
CM-YOLO [19]	89.67	84.16	86.84	89.97	73.17	8.5	3.54
Ours	92.33	87.68	89.94	90.30	73.84	8.0	3.81

**Table 7:** Data composition of the cross-host generalization experiment datasets.

Plant	Class	Train	Val	Test	Objects
Banana	Bract mosaic virus	279	80	40	399
	cordana	391	111	56	558
	healthy	755	216	108	1079
	insectpest	478	139	71	688
	moko	364	98	48	510
	panama	785	224	111	1120
	pestalotiopsis	401	114	58	573
	sigatoka	1106	174	88	1368
	yb_sigatoka	1954	558	280	2792
	Total	6513	1714	860	9087
Groundnut	early leaf spot	702	341	170	1213
	Early rust	1030	294	147	1471
	healthy	286	82	41	409
	late leaf spot	1388	396	200	1984
	nutrition deficiency	1164	333	166	1663
	rust	1205	345	173	1723
	Total	5775	1791	897	8463
Radish	black leaf spot	368	105	53	526
	downey mildew	413	118	60	591
	flea beetle	327	93	47	467
	healthy	425	121	61	607
	mosaic	383	110	55	548
	Total	1916	547	276	2739

**Table 8:** Generalization experiment results.

Plant	Method	Precision (%)	Recall (%)	*F*1-Score	*mAP*50 (%)	*mAP*50-95 (%)	GFLOPs	Params (M)
Banana	YOLOv13n	71.1	70.47	70.78	70.3	53.64	6.3	2.47
	YOLOv12n	73.36	71.59	72.47	74.67	54.58	6.4	2.56
	RDL-YOLO	73.99	73.36	73.68	75.92	56.41	10.4	3.54
	YOLOv11n	73.2	71.93	72.56	74.37	55.61	6.3	2.59
	CM-YOLO	74.07	70.21	72.09	72.54	54.21	8.5	3.54
	YOLOv8n	74.41	69.8	72.04	72.38	54.06	8.1	3.01
	Improved YOLO	71.72	73.11	72.41	74.45	56.88	23.2	8.13
	Ours	72.44	72.67	72.56	73.55	56.55	8	3.82
Groundnut	YOLOv13n	91.97	82.2	85.71	88.69	61.22	6.3	2.47
	YOLOv12n	91.15	81.76	85.75	89.2	62.15	6.4	2.56
	RDL-YOLO	91.6	82.1	84.46	88.55	63.50	10.4	3.54
	YOLOv11n	92.05	82.11	84.97	88.75	62.55	6.3	2.59
	CM-YOLO	91.25	81.93	85.78	88.55	62.10	8.5	3.54
	YOLOv8n	91.49	80.91	84.7	88.37	61.88	8.1	3.01
	Improved YOLO	92.07	84.14	83.55	89.06	63.95	23.2	8.13
	Ours	92.71	82.04	84.66	89	63.85	8	3.82
Radish	YOLOv13n	83.22	88.35	86.81	86.99	45.22	6.3	2.47
	YOLOv12n	85.09	86.41	86.2	88.05	45.66	6.4	2.56
	RDL-YOLO	81.98	87.09	86.61	87.8	46.10	10.4	3.54
	YOLOv11n	83.39	86.62	86.82	87.74	45.88	6.3	2.59
	CM-YOLO	83.6	88.08	86.34	88.94	46.15	8.5	3.54
	YOLOv8n	82.31	87.21	85.87	86.92	45.10	8.1	3.01
	Improved YOLO	80.52	86.82	87.93	87.93	46.33	23.2	8.13
	Ours	81.91	87.59	87.05	88.96	46.50	8	3.82

## Data Availability

The raw data supporting the conclusions of this article will be made available by the authors on request.

## Abbreviations

The following abbreviations are used in this manuscript:

YOLO	You Only Look Once
G-conv	Ghost convolution
ACFFA	Adaptive Calibration and Feature Fusion Architecture Head
AS-WIoU	Adaptive Scale-aware Wise Intersection over Union
Faster R-CNN	Faster Region-based Convolutional Neural Network
CNN	Convolutional Neural Network
PAN	Path Aggregation Network
FPN	Feature Pyramid Network
Conv	Convolution
CBS	Convolution-Batch Normalization-SiLU
C3k2	CSP Stage Block with 3 Convolutions and 2-input Bottleneck
C2f	CSP Stage Block with 2-flows
C2PSA	Cross Stage Partial with Pyramid Squeeze Attention
C3K	CSP Stage Block with 3 Convolutions
AvgPool	Average Pooling
SiLU	Sigmoid Linear Unit
AS	Adaptive Scaling
P	Precision
R	Recall
AP	Average Precision
mAP	Mean Average Precision
GFLOPS	Giga Floating-point Operations Per Second
FPS	Frames Per Second
